# Surgical Approach to Liver Metastasis from Gastroenteropancreatic Neuroendocrine Tumors in the Era of Precision Oncology †

**DOI:** 10.3390/cancers18111745

**Published:** 2026-05-27

**Authors:** Jorgelina Coppa, Simone Oldani, Sara Pusceddu, Monica Paoletti, Marco Bongini, Federica Cavalcoli, Tommaso Cascella, Rodolfo Lanocita, Giovanna Sabella, Massimo Milione, Giovanni Argiroffi, Marco Maccauro, Vincenzo Mazzaferro

**Affiliations:** 1 Hepatology and Hepato-Pancreatic-Biliary Surgery and Liver Transplantation, Fondazione IRCCS Istituto Nazionale Tumori, ENETS Center of Excellence, 20133 Milan, Italy; 2 Department of Medical Oncology, Fondazione IRCCS Istituto Nazionale Tumori, ENETS Center of Excellence, 20133 Milan, Italy; 3 Gastroenterology and Digestive Endoscopy Unit, Fondazione IRCCS Istituto Nazionale Tumori, ENETS Center of Excellence, 20133 Milan, Italy; 4 Department of Radiology, Fondazione IRCCS Istituto Nazionale Tumori, ENETS Center of Excellence, 20133 Milan, Italy; 5 First Pathology Unit, Department of Pathology and Laboratory Medicine, Fondazione IRCCS Istituto Nazionale Tumori, ENETS Center of Excellence, 20133 Milan, Italy; 6 Nuclear Medicine Department, Fondazione IRCCS Istituto Nazionale Tumori, ENETS Center of Excellence, 20133 Milan, Italy

**Keywords:** neuroendocrine tumors, neuroendocrine liver metastases, surgical resection, cytoreductive surgery, liver transplantation, multidisciplinary approach

## Abstract

Neuroendocrine tumors (NETs) are a heterogeneous group of neoplasms with increasing incidence, particularly within the gastroenteropancreatic (GEP) system. The liver represents the most common site of metastasis, and neuroendocrine liver metastases (NELMs) significantly impact prognosis, symptom burden, and therapeutic decision-making. Surgical management remains a cornerstone in the treatment of NELMs and encompasses a spectrum of strategies, including curative liver resection, cytoreductive surgery, and, in selected cases, liver transplantation (LT). Hepatic resection, although potentially curative when technically feasible, is applicable only to a highly selected subset of patients, and its benefits in terms of long-term survival and symptom control remain limited by recurrence rates and patient-related factors. Cytoreductive surgery has emerged as a valuable alternative in patients with unresectable disease, with increasing evidence supporting a ≥70% debulking threshold as sufficient to achieve meaningful clinical benefit. This approach may improve survival and quality of life, notably in symptomatic patients, and can be combined with parenchymal-sparing techniques and locoregional therapies. Liver transplantation represents a radical but potentially curative strategy for highly selected patients with liver-only disease, favorable tumor biology, and stable disease. Outcomes are strongly dependent on strict selection criteria, and appropriate patient selection remains critical. The incorporation of systemic treatments, such as somatostatin analogues, targeted therapies, and peptide receptor radionuclide therapy (PRRT), has broadened the available therapeutic options and contributed to redefining current treatment strategies. Overall, the management of NELMs requires a multidisciplinary, individualized approach guided by tumor biology, disease distribution, and patient-specific factors, with the goal of optimizing survival outcomes and preserving quality of life.

## Simple Summary

Neuroendocrine tumors are rare cancers that often spread to the liver, significantly affecting patient survival and quality of life. Managing liver metastases is challenging because treatment options vary widely and must be tailored to each patient. This review aims to clarify the role of surgery within the broader treatment landscape, including tumor removal, tumor reduction, and liver transplantation in selected cases. It also highlights how newer medical therapies can be combined with surgical strategies to improve outcomes. By summarizing current evidence and treatment approaches, this work seeks to support clinicians in making more informed decisions and to identify areas where further research is needed. Ultimately, improving patient selection and treatment strategies may lead to better long-term survival and quality of life for patients with these complex tumors.

## 1. Introduction

Neuroendocrine tumors (NETs), though historically considered rare, are being diagnosed with increasing frequency worldwide, particularly within the gastroenteropancreatic (GEP) tract. This rise is largely attributed to improvements in diagnostic imaging, endoscopic techniques, and cancer registry reporting [1,2].

The occurrence of metastatic disease, especially involving the liver, is a frequent event and varies according to tumor origin and biological characteristics. Hepatic metastases may be detected at the time of diagnosis in a substantial proportion of patients, with the highest prevalence observed in pancreatic and small bowel tumors. Given the central metabolic role of the liver, the presence of metastases in this organ has major implications for both prognosis and clinical management [3].

Liver involvement represents one of the most relevant prognostic factors in NENs and is typically associated with advanced-stage disease. It often reflects a higher tumor burden, increased biological aggressiveness, and a greater likelihood of complications, particularly in functioning tumors [1]. While patients with localized neuroendocrine tumors (NETs) typically exhibit excellent long-term outcomes, with 10-year survival rates exceeding 90%, the onset of hepatic metastases markedly worsens prognosis, reducing 5-year survival to between 10% and 75%, reflecting the heterogeneity of NETs in terms of grade, differentiation, functionality, and treatment responsiveness [4,5,6].

**Figure 1:**
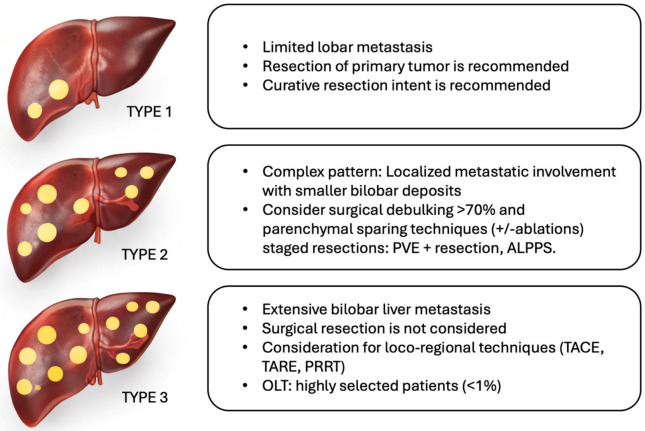
Resectability-oriented classification of NELMs according to anatomical distribution and tumor burden. The figure depicts three patterns of hepatic involvement based on anatomical distribution and tumor burden, with corresponding management strategies ranging from curative-intent resection in limited unilobar disease (Type 1), to parenchymal-sparing and staged surgical approaches in selected bilobar metastases (Type 2), and predominantly loco-regional or transplant-based treatments in extensive bilobar disease in highly selected patients (Type 3). Abbreviations: ALPPS, associating liver partition and portal vein ligation for staged hepatectomy; OLT, orthotopic liver transplantation; PRRT, peptide receptor radionuclide therapy; PVE, portal vein embolization; TACE, transarterial chemoembolization; TARE, transarterial radioembolization.

In recent years, surgical treatment has gained increasing importance in the management of NETs, reflecting a better understanding of disease behavior and therapeutic opportunities. The choice of surgical strategy is influenced by several variables, including the primary tumor site, tumor size, histological grade, and the extent of metastatic spread. These elements are essential in defining both the type of surgical intervention and its role within an overall multidisciplinary treatment plan. The management of NENs with liver metastases requires careful patient selection, individualized therapeutic planning, and integration of surgical and medical strategies within a multidisciplinary framework. A resectability-oriented classification based on lobar involvement may assist in stratifying patients and guiding the selection of surgical, staged, or loco-regional treatment strategies (Figure 1). 

The main topics regarding surgical approaches are outlined below.

## 2. Curative Liver Resection

**Figure 2:**
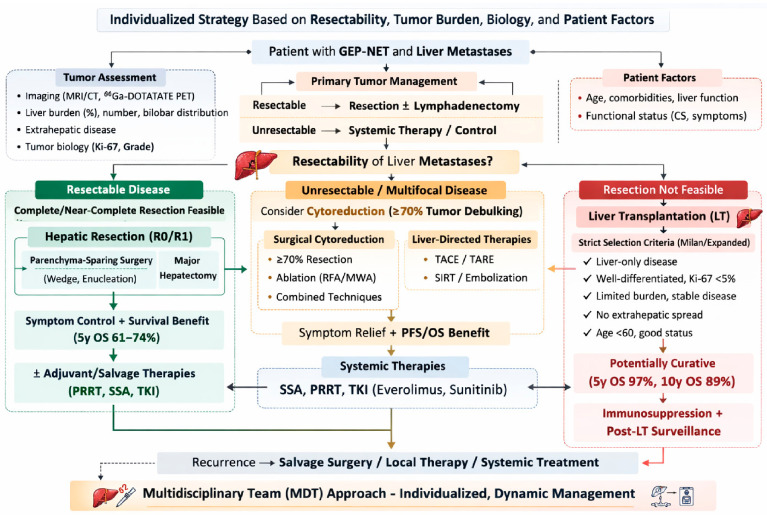
**Algorithm for individualized management of patients with NELMs:** The figure illustrates a resectability-based, multidisciplinary treatment framework integrating tumor burden, biological characteristics, and patient-related factors, with management strategies ranging from curative-intent hepatic resection to cytoreductive, liver-directed, systemic, and transplant-based approaches in selected patients. **Abbreviations:** CS, carcinoid syndrome; GEP-NET, gastroenteropancreatic neuroendocrine tumor; Ki-67, proliferation index; LT, liver transplantation; MDT, multidisciplinary team; MRI, magnetic resonance imaging; OS, overall survival; PET, positron emission tomography; PFS, progression-free survival; PRRT, peptide receptor radionuclide therapy; SSA, somatostatin analogs; SIRT, selective internal radiation therapy; TACE, transarterial chemoembolization; TARE, transarterial radioembolization; TKI, tyrosine kinase inhibitor.

As shown in Figure 2, complete surgical removal of liver metastases should be considered the preferred first-line treatment whenever it is technically achievable. Although the optimal resection margin remains a matter of debate, it is generally accepted that achieving a microscopically negative margin (R0) is associated with improved survival outcomes compared to incomplete resections. Unlike many other metastatic tumors, NELMs often demonstrate an expansive growth pattern with displacement rather than direct infiltration of the surrounding hepatic parenchyma. Consequently, overall survival following R0 (complete) and R1 (microscopically incomplete) resections is often comparable, supporting the consideration of both approaches with curative intent. Despite this potential, only 5–15% of patients are candidates for curative resection, primarily due to the frequent presence of multifocal bilobar disease [7]. Although the optimal width of resection margins remains debated, achieving an R0 resection is generally associated with improved outcomes.

Although numerous studies have examined the role of liver resection in NELMs, the available evidence is largely derived from single-center series with limited sample sizes and marked heterogeneity in patient selection, tumor burden, and treatment intent, substantially limiting the robustness and generalizability of reported outcomes [8]. Moreover, while surgical resection has been associated with prolonged survival and improved control of hormone-related symptoms, including a reduced risk of complications such as carcinoid heart disease, these benefits should be interpreted cautiously, given the noncomparative nature of the data and the rarity of true cure [9]. Reported 5-year overall survival after hepatic resection ranges from 61% to 74% [7,10], compared with 25% to 67% in patients managed with medical therapy alone [4]. However, these wide and overlapping survival estimates likely reflect substantial selection bias, limiting the ability to draw definitive comparative conclusions regarding the true survival advantage of surgical intervention.

Recurrence remains a significant issue affecting most of these patients following surgery [6]. This is partly attributable to preoperative understaging, as NELMs often present with a diffuse, miliary distribution. Pathological analyses have demonstrated that the actual tumor burden may be up to 50% greater than estimated by advanced imaging modalities, including somatostatin receptor scintigraphy, MRI, CT, and ultrasound [11]. Accordingly, reported 5-year recurrence rates reach up to 94% [10] and remain high even after R0 resection (76%) [7].

Comparative studies further support the role of surgery. A systematic review demonstrated significantly improved survival in surgically treated patients, with 5-year OS rates of 60–80% in selected cohorts [12]. Similarly, a meta-analysis including 1108 patients reported superior 1-, 3-, and 5-year OS rates after resection (92.7%, 76.9%, and 67.5%) compared with non-resected patients (77.3%, 40.9%, and 26.6%) [13]. These findings should nonetheless be interpreted with caution, as patients undergoing surgery are typically younger, fitter, and characterized by more biologically favorable disease.

Several clinicopathological factors influence outcomes after liver resection, including primary tumor site, tumor burden (number and size of lesions), and the presence of extrahepatic disease. The largest single-center series, reported by the Mayo Clinic (546 patients, 2000–2020), showed progressive improvements in 5-year OS over time (71%, 78%, and 81%), reflecting advances in patient selection and perioperative management. In multivariable analysis, poorer survival was associated with older age, pancreatic primary tumors, higher Ki-67 index, increased tumor burden, and extrahepatic metastases, with Ki-67 emerging as the strongest prognostic factor. Functional tumors were present in 37% of patients, with symptom relief achieved in 96% of cases [14].

As previously documented, recurrence is frequently observed, particularly among patients presenting with synchronous metastases, extensive hepatic tumor burden, microscopically positive (R1) resections, or those undergoing combined surgical and ablative therapies. Reported recurrence rates range between 50% and 95%, with the liver being the most common site of disease relapse. Factors independently associated with recurrence include a pancreatic primary tumor, lymph node involvement, and positive resection margins. Notably, pancreatic NETs show lower 5-year overall survival rates (30–60%) compared with intestinal NETs (60–90%). Despite this, repeat liver resection can be considered in selected cases of recurrent disease and may be associated with favorable long-term outcomes [15,16,17,18].

The management of high-grade neuroendocrine neoplasms, including neuroendocrine carcinomas (NECs), remains challenging due to their aggressive behavior and the limited availability of robust comparative data. However, selected studies suggest that surgical or ablative approaches may still provide a survival benefit in carefully selected patients, especially when combined with systemic therapies. In a cohort including NECs and well-differentiated G3 NETs, liver resection or radiofrequency ablation (RFA) was associated with a median OS of 35.9 months and a 5-year OS of 43%, with improved outcomes observed in patients with Ki-67 < 55% and those receiving adjuvant chemotherapy [19].

Advanced surgical strategies, such as two-stage hepatectomy and ALPPS, may be considered in selected patient subgroups, particularly in cases of type II hepatic involvement as defined by Frilling et al., characterized by a dominant lesion with bilobar satellite deposits [20,21]. However, ALPPS remains technically demanding and is associated with substantial morbidity, with overall and severe complication rates of 52% and 29%, respectively [21]. Minimally invasive approaches, including laparoscopic and, in selected cases, robotic liver resection, are increasingly favored over open surgery when feasible. These techniques are associated with reduced operative time, lower blood loss, decreased transfusion requirements, and shorter hospital stay [22,23].

## 3. Cytoreduction

Tumor debulking is widely accepted as a key therapeutic strategy in the management of advanced neuroendocrine tumors. However, the optimal extent of cytoreduction in patients with NELMs remains a matter of ongoing debate and continues to evolve [24].

Traditionally, liver-directed surgery was considered appropriate only when at least 90% of the hepatic tumor burden could be removed. Early studies demonstrated that achieving this level of cytoreduction was associated with improved progression-free survival (PFS). For instance, patients undergoing complete (R0) resection showed significantly longer PFS compared with those receiving incomplete resections, despite high recurrence rates. Early evidence supporting this approach was provided by Sarmiento et al., who demonstrated significantly longer PFS in patients undergoing complete (R0) resection compared with incomplete resections, with 5-year recurrence rates of 76% vs. 91% and median PFS of 30 vs. 16 months, respectively (*p* < 0.001) [7]. Similarly, Elias et al. reported 5-year PFS rates of 66%, 46%, and 30% for R0, R1, and R2 resections, respectively, highlighting the prognostic relevance of the extent of cytoreduction [11].

The prognostic role of surgical margin status on long-term outcomes remains a matter of debate. Several studies have not identified a consistent correlation between margin status and patient survival. For example, Mayo et al. found no significant differences in recurrence rates according to margin status, although improved overall survival was observed in patients undergoing R0 or R1 resections compared with those treated with R2 resections [10]. Similar results were reported by Glazer et al. [25]. Likewise, Graff-Baker et al. did not demonstrate a significant disease-specific survival advantage for R0 resections over R2 resections [26]. These heterogeneous findings may be explained by the frequent presence of occult, low-volume disease that is not detected at the time of surgery, even in cases classified as R0 resections. Consequently, the current evidence remains inconsistent, and the true impact of margin status on overall survival is still uncertain.

The North American Neuroendocrine Tumor Society (NANETS) recommends consideration of cytoreductive surgery even in the absence of complete resection, given its potential to improve symptoms and survival outcomes [27].

More recent studies, which should nevertheless be interpreted cautiously, suggest that even lower thresholds of tumor debulking may provide clinically meaningful symptomatic and oncological benefit. In a study conducted by Chambers et al., a reduction in tumor burden of 70% or greater was found to be sufficient for the effective palliation of carcinoid syndrome symptoms [28]. Similarly, Graff-Baker et al. found no significant differences in progression-free or disease-specific survival among patients undergoing 70–89%, 90–99%, or complete (100%) resection, with a median liver PFS of 71.6 months and a 5-year disease-specific survival of 90% [26].

The integration of parenchymal-sparing techniques, including ablation and enucleation, has further facilitated the achievement of these lower debulking thresholds while preserving functional liver parenchyma. In a study of patients with small bowel and pancreatic NELMs, achieving ≥70% cytoreduction was associated with significantly improved PFS (3.2 vs. 1.3 years) and overall survival (OS), whereas increasing the threshold to ≥90% resulted in additional PFS benefit (3.8 vs. 1.5 years) without a statistically significant improvement in OS [16]. These findings were confirmed in an expanded cohort analysis including 188 hepatic cytoreductive procedures. No significant differences in OS or PFS were observed according to the number of metastases treated. However, outcomes were strongly influenced by the degree of cytoreduction: median OS was 37.6 months in patients with <70% debulking, 134.3 months in those with 70–90%, and not reached in patients with >90% cytoreduction (*p* < 0.01 for <70% vs. 70–90%; *p* = 0.6 for 70–90% vs. >90%). Corresponding PFS values were 10.8, 20.6, and 25.3 months, respectively, supporting a clear progression-free survival benefit with increasing tumor reduction [17].

More recent studies have examined the expanded criterion of >70% cytoreduction, especially in pancreatic neuroendocrine tumors (pNETs), applying eligibility criteria that also include patients with intermediate-grade tumors, positive resection margins, or limited extrahepatic disease. In a cohort of 44 patients, no significant difference in progression rates was observed between ≥70% and ≥90% debulking, although median PFS was shorter (11 months), while 5-year OS remained favorable at 81% [29]. Among prognostic factors, only tumor size >5 cm and the use of formal hepatic resection were associated with increased risk of hepatic progression.

Current clinical guidelines recommend considering cytoreductive surgery when complete or near-complete resection is deemed feasible, even in the absence of guaranteed negative margins, provided that at least 70% of the hepatic tumor burden can be removed. This strategy is considered applicable in selected patients with multiple liver lesions or limited extrahepatic disease, particularly in the context of low-aggressive phenotype. However, these recommendations are largely derived from uncontrolled studies and expert consensus, with limited high-level evidence to support a uniform cytoreductive threshold across patient populations. Moreover, the definition of “favorable tumor biology” remains inconsistently applied, and patient selection criteria vary substantially among studies and guidelines. As a result, the generalizability of these recommendations is restricted, and the balance between potential benefit and surgical risk remains uncertain in borderline cases. Accordingly, surgical intervention is generally discouraged in patients with poor performance status, significant comorbidities, advanced liver dysfunction, high-grade tumors, or extensive hepatic involvement where meaningful cytoreduction is unlikely, although the evidence guiding these exclusions is similarly limited and warrants further prospective validation.

## 4. Liver Transplantation

In carefully selected patients with multifocal bilobar disease not amenable to curative resection, liver transplantation (LT) has demonstrated encouraging long-term outcomes. However, due to the rarity and typically indolent course of NELM, robust comparative evidence remains limited, and most available data were derived from registry-based or single institution experiences [30].

Early experience with LT in this setting was associated with variable outcomes, with 5-year OS rates ranging from 40% to 70% and recurrence rates between 31.3% and 56.8% [31,32]. Over time, outcomes have improved, as reflected by data from the European Liver Transplant Registry (ELTR), which reported an increase in 5-year overall survival from 46% before 2000 to 59% in more recent years [33].

These improvements largely reflect refinement of selection criteria and a more nuanced understanding of prognostic factors. A careful balance between potential benefits and inherent risks remains essential, particularly considering long-term survival under immunosuppressive therapy, the risk of disease recurrence, and the persistent limitation of organ availability, which continues to represent a major challenge. Although robust comparative data supporting the use of liver transplantation (LT) in neuroendocrine neoplasms (NENs) remain limited, the accumulated experience from high-volume transplant centers with dedicated programs has contributed to the establishment of evidence-based selection criteria, with the aim of improving outcomes and, ultimately, achieving curative intent. In 2007, Mazzaferro et al. proposed the so-called Milan criteria (Table 1) to define indications for LT in patients with NELM, which are now widely accepted and have informed most transplant programs [34].

Resection of the primary tumor together with regional lymphadenectomy should be performed before considering LT. This approach allows accurate pathological assessment, including evaluation of Ki-67, tumor grading, and staging, facilitates the eradication of extrahepatic disease, and may reduce perioperative risks. Conversely, simultaneous resection of the primary tumor and LT has been associated with worse short- and long-term outcomes [35]. Furthermore, only tumors draining into the portal venous system are considered appropriate candidates, as those with systemic venous drainage carry a higher risk of distant metastases. The presence of extrahepatic disease constitutes an absolute contraindication to LT. Beyond standard pathological parameters, tumor biology can be further assessed through a mandatory observation period of at least 6 months following primary tumor resection. This “waiting period” acts as a surrogate indicator of tumor aggressiveness, allowing early identification of rapidly progressive disease and reducing the so-called “fast-track” effect, defined as premature transplantation in patients at risk of post-LT recurrence [36,37]. Tumor burden is another key prognostic factor. Hepatic involvement exceeding 50% has consistently been associated with poorer outcomes following transplantation [38]. Similarly, hepatomegaly, defined as an explanted liver volume exceeding the standard volume by more than 20%, has been identified as an independent predictor of unfavorable prognosis [31]. In addition, younger age (<60 years) should be considered a relative selection criterion, as long-term benefits of LT tend to decrease with advancing age [31,36] (Table 1).

**Table 1:** Selection criteria for orthotopic liver transplantation (OLT) for NELMs.

	Milan Criteria [34]	UNOS/OPTN 2 [39]	ENETs [40]	NANETs [41]
**Histology**	G1–G2, Mib 1 < 10%	G1–G2	G1–G2	NA
**Primary tumor site**	Portal system drainage	Portal system drainage	NA	
**Liver involvement**	<50% of liver volume	<50%of liver volume	NA	NA
**Time interval of stable disease**	>6 months	>6 months prior to MELD exception request	NA	NA
**Recipient age**	<60 years (relative criteria)	<60 years	NA	NA
**Other**	Extended Milan criteria < 70 years	GEP origin, liver extension non-resectable Negative metastatic work up	Functional NETs and diffuse liver disease, refractory to multiple systemic therapies No extrahepatic disease	OLT is controversial, but may be an option if the Milan and Enets criteria are met

**Abbreviations:** ENETS, European Neuroendocrine Tumor Society; GEP, gastroenteropancreatic; MELD, Model for End-Stage Liver Disease; NANETS, North American Neuroendocrine Tumor Society; NET, neuroendocrine tumor; OLT, orthotopic liver transplantation; OPTN, Organ Procurement and Transplantation Network; UNOS, United Network for Organ Sharing.

Under these stringent criteria, LT has achieved excellent long-term outcomes, with reported 5- and 10-year OS rates of 97% and 89%, respectively, along with a significant transplant-related survival benefit [34]. Accordingly, liver transplantation, when integrated with perioperative multimodal medical therapy, may offer a potentially curative option for carefully selected patients with NELM. Several transplant programs have developed specific criteria to identify appropriate candidates, most of which are based on the previously described selection principles. In the United States, these criteria have been adopted by the Organ Procurement and Transplantation Network (OPTN), enabling eligible patients with NELM to be listed under MELD exception status [39].

According to the European Neuroendocrine Tumor Society (ENETS), LT may be considered a salvage option in carefully selected patients, particularly those with functional tumors or carcinoid syndrome and extensive hepatic involvement who have failed multiple systemic therapies [40]. The North American Neuroendocrine Tumor Society (NANETS) considers LT a controversial option but supports the use of Milan criteria and ENETS recommendations for patient selection [27,41] (Table 1).

A prospective clinical trial would be the most appropriate approach to better clarify the role of liver transplantation in patients with NELMs and could become feasible once adequate expertise and organizational infrastructure are established to support patient identification and enrollment.

Comparative studies have suggested a survival advantage of LT over liver resection (LR) in patients meeting Milan criteria. Long-term results from the Mazzaferro group showed higher OS and disease-free survival (DFS) in transplant recipients compared with resected patients, with longer disease-free intervals (78 vs. 24 months) and different recurrence patterns, with multisite recurrence more common after LT and intrahepatic recurrence after LR. A propensity score-matched analysis further supported these findings, demonstrating improved long-term survival and disease control in the LT group [42].

More recently, Eshmuminov et al. conducted a multicenter study including 455 patients, showing significantly longer median OS in the LT group compared with R0 resection (205 vs. 120 months), with 5-year OS rates of 75% and 68.3%, respectively (*p* = 0.015). This benefit was particularly evident in patients within Milan criteria and with indolent disease biology (G1, Ki-67 < 5%), whereas no advantage was observed outside these criteria, underscoring the critical importance of appropriate patient selection [43].

Despite these promising outcomes, several unresolved challenges remain. These include defining the optimal timing of LT within the disease course, clarifying its incremental benefit in the era of effective systemic therapies such as peptide receptor radionuclide therapy (PRRT), and determining the role of living donor liver transplantation (LDLT) in expanding access to treatment. Ongoing debates also concern whether selection criteria could be safely expanded to include patients with higher tumor burden, functional syndromes, or more advanced age. The potential role of ^177^Lu-labeled radioligand therapy as both a competing therapeutic option and a neoadjuvant or adjuvant strategy further complicates the therapeutic landscape. In this setting, prospective clinical trials would be essential to better define the role of LT in patients with NELM and to optimize its integration into multimodal treatment strategies.

## 5. Neoadjuvant Systemic Treatments

In patients with neuroendocrine tumors (NETs) and liver metastases, neoadjuvant systemic therapy may be considered a selective strategy aimed at tumor downsizing, biological patient selection, and the potential conversion to liver-directed surgical approaches. This strategy is particularly relevant in pancreatic NETs (pNETs), in which objective radiological responses are more frequently observed than in small-intestinal NETs. In this setting, systemic treatment may significantly influence surgical feasibility, especially in patients with borderline resectable disease or liver-dominant tumor burden [2,44]. Among available systemic approaches, CAPTEM currently represents the most convincing cytotoxic regimen in terms of cytoreduction. The randomized ECOG-ACRIN E2211 trial demonstrated improved progression-free survival and overall survival with CAPTEM compared with temozolomide alone in advanced pNETs, while also confirming clinically meaningful objective response rates, thereby supporting its use when cytoreduction is required before liver surgery or combined liver-directed procedures [45]. Consequently, CAPTEM is increasingly regarded as a potential conversion strategy in patients with high hepatic tumor burden, bilobar disease, or situations in which downsizing could facilitate R0/R1 resection, parenchymal-sparing hepatectomy, or combined ablation-resection approaches [46].

In parallel, peptide receptor radionuclide therapy (PRRT) has been proposed as a potential neoadjuvant or conversion approach in selected patients with somatostatin receptor (SSTR)–positive NETs, particularly in those with relatively indolent tumor biology, liver-predominant disease, and high SSTR expression on molecular imaging [47]. Although the NETTER-1 trial established ^177^Lu-DOTATATE as an effective therapy in progressive midgut NETs through substantial improvement in progression-free survival and durable disease control, its relevance in the neoadjuvant setting derives from the capacity of PRRT to induce prolonged stabilization and, in selected patients, sufficient tumor regression to allow subsequent surgical or locoregional treatment [47]. Recent reviews and ENETS guidance papers have increasingly emphasized the possibility of integrating PRRT earlier in the therapeutic sequence, including as a preoperative strategy in carefully selected patients with NELM [2]. In addition to tumor shrinkage, PRRT may provide important biological information by identifying patients with aggressive disease progression despite receptor positivity, thereby helping to avoid futile major hepatectomy. Combination approaches integrating PRRT with radiosensitizing chemotherapy, particularly capecitabine or temozolomide, are also under active investigation and may further improve response rates in pancreatic NETs, although prospective evidence remains limited [48].

Conversely, the role of targeted biological therapies, such as everolimus and sunitinib, in the neoadjuvant setting appears less well established. Both agents have demonstrated relevant antiproliferative activity in advanced, well-differentiated NETs, leading to significant improvements in progression-free survival in pivotal randomized trials, including RADIANT-3 and RADIANT-4 for everolimus and the phase III trial of sunitinib in advanced pancreatic NETs [49,50,51]. However, objective response rates have consistently remained low, suggesting that these treatments predominantly result in disease stabilization rather than substantial radiological tumor shrinkage. As a result, everolimus and sunitinib are generally regarded as less suitable than CAPTEM or PRRT when the primary therapeutic goal is conversion to resectability or major cytoreduction of liver metastases. Their potential role may instead be confined to selected patients with biologically indolent tumors, in whom disease stabilization prior to surgery is considered desirable, or to patients who are not candidates for chemotherapy or PRRT [52,53].

Overall, neoadjuvant systemic therapy appears most clinically valuable when a radiological or biological response would directly alter management, for example, by enabling liver resection, staged hepatectomy, parenchymal-sparing surgery, or integrated ablative strategies. Nevertheless, the available evidence remains largely non-uniform and based on extrapolation from advanced-disease studies rather than dedicated neoadjuvant trials. Current data therefore support a highly individualized multidisciplinary approach, particularly in well-differentiated pNETs treated with CAPTEM and in SSTR-positive NETs treated with PRRT, where the probability of meaningful tumor reduction and improved surgical selection appears greatest [2,46,48].

Systemic therapy should no longer be regarded only as a palliative intervention, but increasingly as a dynamic tool for biological selection, therapeutic stratification, and optimization of candidacy for aggressive liver-directed strategies.

From a practical perspective, treatment sequencing should increasingly be guided by both technical resectability and dynamic biological behavior. In patients with borderline resectable or extensive liver-dominant disease, CAPTEM may be preferentially considered in pancreatic NETs requiring objective volumetric reduction, whereas PRRT may be particularly valuable in SSTR-positive tumors characterized by indolent but diffuse hepatic involvement. In contrast, liver transplantation should remain restricted to highly selected patients with sustained disease stability, a favorable biological profile, and the absence of extrahepatic progression despite multimodal therapy. Within this framework, systemic therapy may serve not only as treatment but also as a dynamic tool for biological selection prior to aggressive liver-directed strategies.

## 6. Genomic Profiling and Molecular Biomarkers in Surgical Selection

The increasing availability of molecular profiling has progressively improved the understanding of the biological heterogeneity of GEP-NETs. Although genomic biomarkers are not currently incorporated into formal surgical selection criteria for liver resection or LT, accumulating evidence suggests that molecular characterization may contribute to refining prognostic stratification and identifying patients with more favorable tumor biology. Currently, in clinical practice, selection for hepatic resection or LT still relies predominantly on conventional clinicopathological parameters, including tumor grade, Ki-67 index, hepatic tumor burden, absence of extrahepatic disease, and longitudinal disease stability, as reflected by Milan, ENETS, and UNOS criteria [42,43]. However, the concept that “tumor biology cuts the deal” has become increasingly relevant in the management of NELMs, particularly in borderline surgical scenarios.

Among the most extensively investigated biomarkers in pNETs, alterations involving DAXX and ATRX, together with the alternative lengthening of telomeres (ALT) phenotype, appear to carry important prognostic implications. Loss of DAXX/ATRX expression and ALT positivity have consistently been associated with metastatic dissemination, increased risk of recurrence, and inferior survival outcomes, even in well-differentiated tumors [54,55]. These findings suggest that molecular profiling may help identify biologically aggressive neoplasms that are technically resectable but potentially less likely to benefit from highly aggressive liver-directed strategies. Conversely, tumors lacking these high-risk molecular features may exhibit a more indolent clinical course and potentially more favorable long-term outcomes after surgery or transplantation.

Liquid biopsy approaches have also emerged as promising tools for dynamic disease monitoring. In particular, the NETest, a multigene circulating mRNA assay, has demonstrated encouraging prognostic performance in patients undergoing surgical treatment for NETs. Persistent postoperative NETest positivity has been associated with early recurrence after apparently curative resection, whereas normalization of NETest levels correlates with prolonged disease-free survival [56,57]. Although these assays are not yet routinely integrated into clinical decision-making algorithms, they may eventually contribute to postoperative surveillance strategies and improve biological selection for repeat liver-directed interventions or transplantation.

Molecular profiling may additionally influence therapeutic sequencing in advanced disease. Recent evidence suggests that mutations involving MEN1, DAXX, and ATRX may correlate with improved progression-free survival in patients treated with peptide receptor radionuclide therapy (PRRT), raising the possibility that genomic profiling could help identify patients more likely to achieve durable disease control before surgical reconsideration [58]. This concept may be particularly relevant in patients initially considered unsuitable for resection or LT, in whom systemic therapy is used as a biological selection strategy prior to aggressive surgical approaches.

Despite these promising developments, the integration of genomic data into surgical decision-making algorithms for neuroendocrine liver metastases (NELMs) remains investigational. The currently available evidence is largely retrospective and inconsistent, and no molecular signature has yet achieved sufficient validation to independently inform indications for liver resection or transplantation. Nevertheless, the progressive incorporation of tissue-based genomics, liquid biopsy approaches, molecular imaging, and dynamic assessment of treatment response may, over time, enable a more individualized and biologically driven framework for the surgical management of metastatic NETs.

## 7. Practical Limitations and Unmet Needs in Real-World Management

As a summary, Table 2 aims to not only highlight and outline the current guideline, but also summarize the main clinical practice currently adopted daily regarding the surgical management of NELMs. The guidelines primarily represent professional agreement rather than high-level prospective data, despite their attempt to outline important selection criteria for the various surgical procedures for NELMs. Therefore, rather than offering final, evidence-based treatment protocols, they offer a conceptual framework for patient categorization, highlighting the need for more recent, methodologically sound research to improve surgical decision making in this context. Many of these recommendations are based on somewhat out-of-date research.

Despite a comprehensive review of the available literature and a systematic discussion of surgical approaches for NENLMs, several important limitations of the present work must be acknowledged. First, the evidence underpinning most surgical recommendations is largely derived from non-randomized, single-center series or registry-based studies. These analyses are often limited by small sample sizes, different inclusion criteria, inconsistent definitions of resectability, and non-standardized treatment strategies, all of which compromise the robustness and reproducibility of the reported outcomes. Most notably, the absence of prospective, randomized trials significantly restricts the overall level of evidence guiding current clinical decision-making. Furthermore, surgical series are inherently prone to selection bias. This largely reflects the absence of validated tools for dynamic treatment allocation over time. In routine clinical practice, therapeutic strategies are frequently adapted iteratively; however, no standardized framework currently exists to guide transitions among surgical, locoregional, and systemic treatments. As a result, patients selected for liver surgery or liver transplantation are typically younger, have better performance status, more favorable tumor biology, and limited extrahepatic disease. Consequently, the survival advantages reported after surgical intervention cannot be definitively attributed to the procedures themselves but may instead reflect the intrinsically better prognosis of the selected patient populations. Another important unmet need concerns the definition of optimal treatment sequencing. While multiple options are available, the timing of surgery in relation to systemic therapies as neoadjuvant intent remains largely empirical and is influenced by institutional experience rather than high-level evidence. The role of surgery in borderline or atypical scenarios also remains unclear, including patients with limited extrahepatic disease, intermediate-grade tumors, or mixed response to systemic treatments. These situations are frequently encountered in practice but are underrepresented in the literature.

NETs represent a highly heterogeneous disease spectrum, encompassing tumors that vary markedly in terms of primary site, differentiation grade, proliferative index (Ki-67), functional status, and growth kinetics. This biological heterogeneity limits the applicability of uniform surgical thresholds, such as fixed cytoreduction percentages, across diverse patient subgroups and complicates the interpretation and generalization of pooled survival outcomes. The prognostic significance of surgical margin status remains controversial. While microscopically negative resections (R0) are generally associated with improved outcomes, several studies have reported comparable overall survival between R0 and R1 resections. This inconsistency likely reflects the frequent presence of occult microscopic disease not detectable by current imaging techniques, even in cases deemed completely resected, thereby limiting the clinical relevance of margin status as an isolated prognostic indicator. Recurrence rates following hepatic resection remain remarkably high, even after apparently curative surgery. This raises important questions regarding the true curative potential of liver surgery and underscores the need for long-term surveillance and multimodal treatment strategies. The high incidence of post-operative recurrence further complicates comparisons between surgical and non-surgical approaches.

Finally, real-world management is often constrained by organizational and logistical factors, including variability in referral pathways and access to highly specialized procedures, which may influence treatment opportunities independently of tumor characteristics.

The impact of evolving systemic therapies on long-term surgical outcomes has not been fully established. In particular, how prior or subsequent treatments may influence resectability, recurrence patterns, and survival requires further investigation. There is also a need for better integration of longitudinal disease monitoring into clinical decision-making. Current approaches do not fully incorporate changes in tumor behavior over time, which may be relevant in a disease characterized by variable growth kinetics.

Greater emphasis should be placed on refining patient selection through improved characterization of tumor biology. The integration of molecular and genomic profiling, functional imaging parameters, and dynamic biomarkers may allow more accurate stratification of patients according to biological aggressiveness and expected benefit from surgery. In this context, Ki-67 alone appears insufficient to fully capture tumor behavior, and composite prognostic models incorporating molecular, radiological, and clinical variables warrant further investigation. The establishment of international registries and collaborative networks will be critical to advancing the field. Given the rarity and heterogeneity of NENs, large-scale data sharing is essential to generate sufficiently powered analyses, validate prognostic models, and translate emerging biological insights into clinically meaningful surgical strategies.

The optimal integration of surgery within modern multimodal treatment algorithms also remains an important area for future study. Advances in systemic therapies, like PRRT, targeted agents, and emerging radioligand treatments, raise critical questions regarding the timing and sequencing of surgical intervention. Prospective studies evaluating surgery as part of combined or neoadjuvant approaches, as well as its role following treatment response or disease stabilization, are needed to define its incremental benefit in the contemporary therapeutic landscape.

Although liver transplantation has demonstrated excellent long-term outcomes in highly selected patients, its general applicability is constrained by stringent selection criteria, limited organ availability, and the absence of prospective comparative trials. As such, the role of transplantation remains restricted to specialized centers and carefully chosen clinical scenarios. The potential role of living donor liver transplantation, as well as neoadjuvant or bridging therapies such as PRRT, deserves further exploration in carefully designed studies.

In clinical practice, treatment sequencing in patients with NELMs should be individualized according to disease distribution, tumor biology, and response dynamics. Patients with limited, technically resectable liver-confined disease and indolent tumor behavior should generally be considered for upfront hepatic resection. In contrast, patients with borderline resectable pancreatic NETs, high hepatic tumor burden, or bilobar involvement may benefit from an initial chemotherapy-based approach when meaningful tumor shrinkage is required to facilitate parenchymal-sparing surgery or combined resection-ablation strategies. For patients with diffuse but strongly SSTR-positive liver-dominant disease, PRRT may represent a more appropriate first therapeutic step, particularly when prolonged disease stabilization rather than rapid cytoreduction is the primary objective. In this setting, the response to systemic therapy may also provide important information regarding underlying tumor biology and help refine subsequent surgical decision-making. Conversely, rapid progression despite systemic treatment should raise caution regarding aggressive liver-directed procedures, including transplantation, given the higher likelihood of biologically unfavorable disease. Liver transplantation should therefore remain reserved for highly selected patients with sustained liver-only disease control, low-grade histology, and favorable longitudinal biological behavior despite prolonged observation and multimodal treatment.

## 8. Conclusions

The management of NELMs is increasingly driven by tumor biology and multimodal integration rather than surgical feasibility alone. Hepatic resection remains the cornerstone of treatment and should be pursued whenever feasible, while cytoreductive surgery extends surgical benefit to a broader population when adequate tumor debulking can be achieved. Liver transplantation offers a potentially curative option, but its role is restricted to rigorously selected patients and remains limited by strict eligibility criteria and organ availability.

Despite these advances, the high rate of recurrence and the growing complexity of therapeutic options highlight the systemic nature of NELMs and the need for optimized multimodal approaches. In particular, the expanding use of systemic therapies, especially PRRT and CAPTEM, is reshaping treatment algorithms and emphasizing the importance of treatment sequencing, which remains inadequately defined.

Future progress will depend on prospective, multicenter collaborative efforts aimed at refining patient selection, standardizing definitions of resectability and treatment endpoints, and integrating dynamic biomarkers, molecular profiling, and longitudinal disease assessment into clinical decision-making. Particular emphasis should be placed on clarifying the role and timing of surgery within modern systemic treatment pathways, including neoadjuvant and conversion strategies.

Ultimately, a personalized, integrated treatment strategy remains essential to maximize outcomes in patients with NELMs, while ongoing research will be critical to better define the role of surgery in the evolving therapeutic landscape. The future of surgical management of NELMs will likely depend less on technical feasibility alone and increasingly on the integration of dynamic biomarkers, molecular profiling, and longitudinal assessment of tumor biology.

## Figures and Tables

**Table 2:** Comparative Overview of Surgical Guidelines recommendations for Advanced Neuroendocrine NETs.

Surgical Aspect	ENETS [40]	NANETS [41]	ESMO [59]
**Liver Surgery** **(R0 Resection)**	Hepatic resection can be performed either in a single procedure or in staged approaches. When surgery is not feasible, locoregional treatments such as RFA, TACE, or SIRT should be considered	Liver resection may improve both hormonal symptom control and survival outcomes and should be considered when technically feasible and associated with acceptable morbidity and mortality	Surgical treatment of liver disease is recommended in patients with uncontrolled functional tumors, provided that at least 70% of tumor burden can be removed
**Liver Debulking** **Surgery**	May be considered in patients with functional tumors that are not adequately controlled with medical therapy	Debulking surgery is appropriate if ≥70% of tumor burden can be safely removed	Similar indications apply, particularly in patients with hormonally active disease
**Liver Transplantation**	Considered only in highly selected patients (approximately 1%) who are refractory to other therapeutic options	LT remains a debated option, but may be considered in patients fulfilling Milan and ENETS selection criteria	May be considered in exceptional cases meeting strict eligibility requirements
**Adjuvant/Neoadjuvant** **Therapy**	NA	NA	NA

**Abbreviations:** NETs: Neuroendocrine tumors; ENETS: European Neuroendocrine Tumor Society; NANETS: North American Neuroendocrine Tumor Society; ESMO: European Society for Medical Oncology; R0: Complete surgical resection with negative margins; RFA: Radiofrequency ablation; TACE: Transarterial chemoembolization; SIRT: Selective internal radiation therapy; LT: Liver transplantation; NA: not available.

## Data Availability

No new data were created or analyzed in this study. Data sharing is therefore not applicable.

## Abbreviations

The following abbreviations are used in this manuscript:

NETs	neuroendocrine tumors
SEER	Surveillance, Epidemiology, and End Results Program
NENs	neuroendocrine neoplasms
GEP-NET	gastroenteropancreatic neuroendocrine tumors
GEP	gastroenteropancreatic
NECs	neuroendocrine carcinomas
WHO	World Health Organization
SSRTs	somatostatin receptors
PRRT	peptide receptor radionuclide therapy
NELMs	neuroendocrine liver metastases
pNETs	pancreatic neuroendocrine neoplasms
OS	overall survival
PFS	progression-free survival
DFS	disease-free survival
RCTs	randomized trials
TTP	time to progression
ALPPS	Associating Liver Partition and Portal Vein Ligation for Staged Hepatectomy
OLT	orthotopic liver transplantation
LT	liver transplantation
SB-NETs	small bowel neuroendocrine tumors
CHD	carcinoid heart disease
CS	carcinoid syndrome
SSAs	somatostatin analogues
mTOR	mammalian target of rapamycin
VEGF	vascular endothelial growth factor
LRTs	locoregional therapies
SIRT	selective internal radiation therapy
TARE	transarterial radioembolization
MDT	multidisciplinary team
MELD	model of End-Stage liver Disease
ESMO	European Society for Medical Oncology
UNOS/OPTN	united network for organ sharing/organ procurement and transplantation Network
ENETs	European Neuroendocrine Tumor Society
NANETs	North America Neuroendocrine Tumor Society
PVE	portal vein embolization
